# Prospective cohort of cryobiopsy in interstitial lung diseases: a single center experience

**DOI:** 10.1186/s12890-022-01990-4

**Published:** 2022-06-02

**Authors:** Manuel L. Ribeiro Neto, Andrea Valeria Arrossi, Ruchi Yadav, Daniel A. Culver, Sanjay Mukhopadhyay, Joseph G. Parambil, Brian D. Southern, Leslie Tolle, Aman Pande, Francisco A. Almeida, Debasis Sahoo, Jessica Glennie, Usman Ahmad, Atul C. Mehta, Thomas R. Gildea

**Affiliations:** 1 Department of Pulmonary Medicine, Respiratory Institute, Cleveland Clinic, 9500 Euclid Avenue / A90, Cleveland, OH 44195 USA; 2 Department of Pathology, Cleveland Clinic, Cleveland, USA; 3 Section of Thoracic Imaging, Imaging Institute, Cleveland Clinic, Cleveland, USA; 4 Thoracic Surgery Department, Heart, Vascular and Thoracic Institute, Cleveland Clinic, Cleveland, USA

**Keywords:** Bronchoscopy, Biopsy, Diagnosis, Hemorrhage, Learning curve

## Abstract

**Rationale:**

Transbronchial cryobiopsy has been increasingly used to diagnose interstitial lung diseases. However, there is uncertainty regarding its accuracy and risks, mainly due to a paucity of prospective or randomized trials comparing cryobiopsy to surgical biopsy.

**Objectives:**

To evaluate the diagnostic yield and complications of cryobiopsy in patients selected by multidisciplinary discussion.

**Methods:**

This was a prospective cohort from 2017 to 2019. We included consecutive patients with suspected interstitial lung diseases being considered for lung biopsy presented at our multidisciplinary meeting.

**Measurements and main results:**

Of 112 patients, we recommended no biopsy in 31, transbronchial forceps biopsy in 16, cryobiopsy in 54 and surgical biopsy in 11. By the end of the study, 34 patients had had cryobiopsy and 24 patients, surgical biopsy. Overall pathologic and multidisciplinary diagnostic yield of cryobiopsy was 47.1% and 61.8%, respectively. The yield increased over time for both pathologic (year 1: 28.6%, year 2: 54.5%, year 3: 66.7%, *p* = 0.161) and multidisciplinary (year 1: 50%, year 2: 63.6%, year 3: 77.8%, *p* = 0.412) diagnosis. Overall rate of grade 4 bleeding after cryobiopsy was 11.8%. Cryobiopsy required less chest tube placement (11.8% vs 100%, *p* < 0.001) and less hospitalizations compared to surgical biopsy (26.5% vs 95.7%, *p* < 0.001), but hospitalized patients had a longer median hospital stay (2 days vs 1 day, *p* = 0.004).

**Conclusions:**

Diagnostic yield of cryobiopsy increased over time but the overall grade 4 bleeding rate was 11.8%.

**Supplementary Information:**

The online version contains supplementary material available at 10.1186/s12890-022-01990-4.

## Introduction

Transbronchial cryobiopsy (TBCB) has been increasingly used in the diagnostic evaluation of patients with interstitial lung diseases (ILD) [1]. Since one of the first published experiences more than a decade ago [2], many centers around the world have adopted and described their outcomes with TBCB in ILD [3–6]. More recently, expert guidelines have been published in an effort to standardize the technique and optimize the risk–benefit ratio to patients [7, 8].

The first prospective study evaluating diagnostic yield on sequential TBCB and surgical lung biopsy (SLB) was the CryoPID study [9]. The investigators performed both TBCB and SLB during the same procedure in 21 patients and found poor pathologic concordance between the techniques (agreement 38%, kappa 0.22). However, a more recent prospective cohort in Australia (COLDICE) found different results [10]. In this study, 65 patients with ILD had sequential TBCB and SLB during a single procedure after screening through a multidisciplinary discussion (MDD). The biopsy samples were allocated in a random sequence, evaluated by pathologists blinded to the clinical and imaging findings, and finally discussed at MDD in a de-identified fashion. A pathologic diagnosis was achieved in 90.7% of TBCB and 96.9% of SLB. Agreement between TBCB and SLB was 70.8% (kappa 0.70) between pathologists and 76.9% (kappa 0.62) between MDD groups. However, in both CryoPID and COLDICE, a comparison of risks from each procedure was not possible due to the study design.

The risks of TBCB have been evaluated in multiple cohort studies and meta-analyses. In the largest cohort to date from Italy, the incidence of pneumothorax post TBCB in 699 patients was 19.2%. The overall incidence of bleeding was 12.4% (7.6% moderate and 0.7% severe) and 30-day mortality was 0.4% [11]. The incidence of moderate/severe bleeding was higher (12%) in a smaller cohort from the United States [12] and in two meta-analyses: 20.1% (95% CI 5.6–42.8) in one [4] and 26.6% (range 0–78) in another [6]. A recent letter to the editor contributed to this debate, by showing pictures taken during video-assisted thoracoscopic surgeries (VATS) immediately after TBCB. The images demonstrate wounds and hematomas inflicted by TBCB [13].

The comparison of risks between TBCB and transbronchial forceps biopsy (TBFB) have been studied in randomized controlled trials (RCT). A Spanish RCT including 77 patients showed an incidence of moderate bleeding of 56.4% in TBCB versus 34.2% in TBFB [14]. A German RCT including 359 patients showed bleeding incidences of 15% (moderate) and 1.1% (severe) in TBCB versus 4.2% (moderate) and 0% (severe) in TBFB. [15].

The literature comparing risks between TBCB and SLB, however, lacks direct comparisons by prospective or randomized trials. One Italian retrospective cohort including 150 patients who had SLB and 297 patients who had TBCB found no severe bleeding in either group. Compared to TBCB, SLB was associated with a longer hospital stay (median 6.1 vs 2.6 days, *p* < 0.001) and higher 60-day mortality (2.7% vs 0.3%, *p* = 0.045) [3].

We implemented a prospective cohort study in our institution to evaluate the outcomes of TBCB. Our primary aim was to evaluate the diagnostic yield and complications of TBCB in patients selected by MDD in a large academic center with widely available expertise in interstitial lung diseases. A secondary aim was to compare outcomes between TBCB and SLB.

## Methods

### Study design and population

This was a prospective cohort study at a single institution from 2017 to 2019. We included consecutive patients with suspected ILD being considered for lung biopsy presented at our MDD. This study was approved by the Cleveland Clinic Institutional Review Board (number 16-1712). A waiver of informed consent was approved due to this being a minimal risk study using data collected for routine clinical practice. All methods were performed in accordance with the Declaration of Helsinki.

### Study procedures

We reviewed clinical, radiological, and prior pathologic data (if available) during MDD and recorded the radiological pattern and most likely diagnosis. The radiological and pathologic diagnoses were recorded prospectively according to the American Thoracic Society (ATS) 2011 Idiopathic Pulmonary Fibrosis (IPF) guidelines [16] and later reclassified retrospectively according to the ATS 2018 IPF guidelines [17]. Per the guidelines, usual interstitial pneumonia (UIP) histopathology features are dense fibrosis with architectural distortion (i.e., destructive scarring and/or honeycombing), predominant subpleural and/or paraseptal distribution of fibrosis, patchy involvement of lung parenchyma by fibrosis, fibroblast foci, and absence of features to suggest an alternate diagnosis. The MDD generated 4 different recommendations: no biopsy, TBFB, TBCB, or SLB. No prespecified criteria were used to guide these recommendations. The recommendations were based on a live discussion considering multiple factors, as usual in our clinical practice. If TBCB was recommended, the best segments(s) for biopsy were selected during MDD. All TBCB cases were discussed in the MDD post procedure. Genomic classifier (Envisia®, Veracyte) became available in our institution in April 2019.

### Cryobiopsy protocol

Before every procedure we tested in normal saline the freezing time required to achieve a biopsy sample diameter of approximately 5 mm. All cryobiopsies were performed under general anesthesia and rigid bronchoscopy according to previously published guidelines [8]. Bronchioalveolar lavage (BAL) was performed in all patients. We added a 3-min ipsilateral mainstem occlusion with an Arndt bronchial blocker (Cook® Medical) to assess the patient’s tolerability to single lung ventilation. The procedure was aborted if the SpO2 dropped by > 4%. We positioned the bronchial blocker closer to the take-off of the segment of interest maintaining at least lobar isolation. Under fluoroscopy guidance, a 1.9 mm cryoprobe (Erbe®, item number 20416-036, length 900 mm) was advanced either to the fluoroscopic pleural line or until resistance was met and then retracted by 1 cm, where the freezing cycle was initiated. Once the freezing cycle was completed, the flexible bronchoscope and the cryoprobe were removed from the airway *en bloc*, and the bronchial blocker was immediately inflated for 4 min after each cryobiopsy. Once all samples were obtained and no active bleeding was present, the rigid bronchoscope was replaced by a laryngeal mask and fluoroscopy was used to evaluate for pneumothorax. The bronchoscopy team was comprised of an interventional pulmonologist (IP), a non-IP bronchoscopist, and an IP fellow. These three bronchoscopists alternated cryobiopsy passes in the same procedure.

### Outcome measurements

We collected data on intraoperative variables (procedure duration, time in positive pressure, time with FiO_2_ of 100%, fluid balance), cryobiopsy largest diameter, pathologic diagnosis, MDD consensus diagnosis, early complications (bleeding, pneumothorax, pneumomediastinum, duration of chest tube), and complications within 30 days (hospitalization, mechanical ventilation, pneumonia, acute exacerbation, death). Bleeding and pneumothorax severity were classified according to the Common Terminology Criteria for Adverse Events as previously described [18]. Grade 4 bleeding was defined as respiratory compromise requiring intubation.

### Statistical analysis

Descriptive statistics were used to summarize the data. Continuous variables were summarized as mean ± standard deviation or median with p25–p75 or range. Categorical variables were summarized as absolute and relative frequencies. Due to the small sample size, continuous variables were compared with a non-parametric test (Mann Mann–Whitney U test or Kruskal–Wallis test) and no multivariable analyses were performed. Categorical variables were compared with Fisher’s Exact test or Pearson’s Chi Square test. Data were stored in REDCap® and analyzed in SPSS® version 21.

## Results

### Study overview and baseline characteristics

**Fig. 1:**
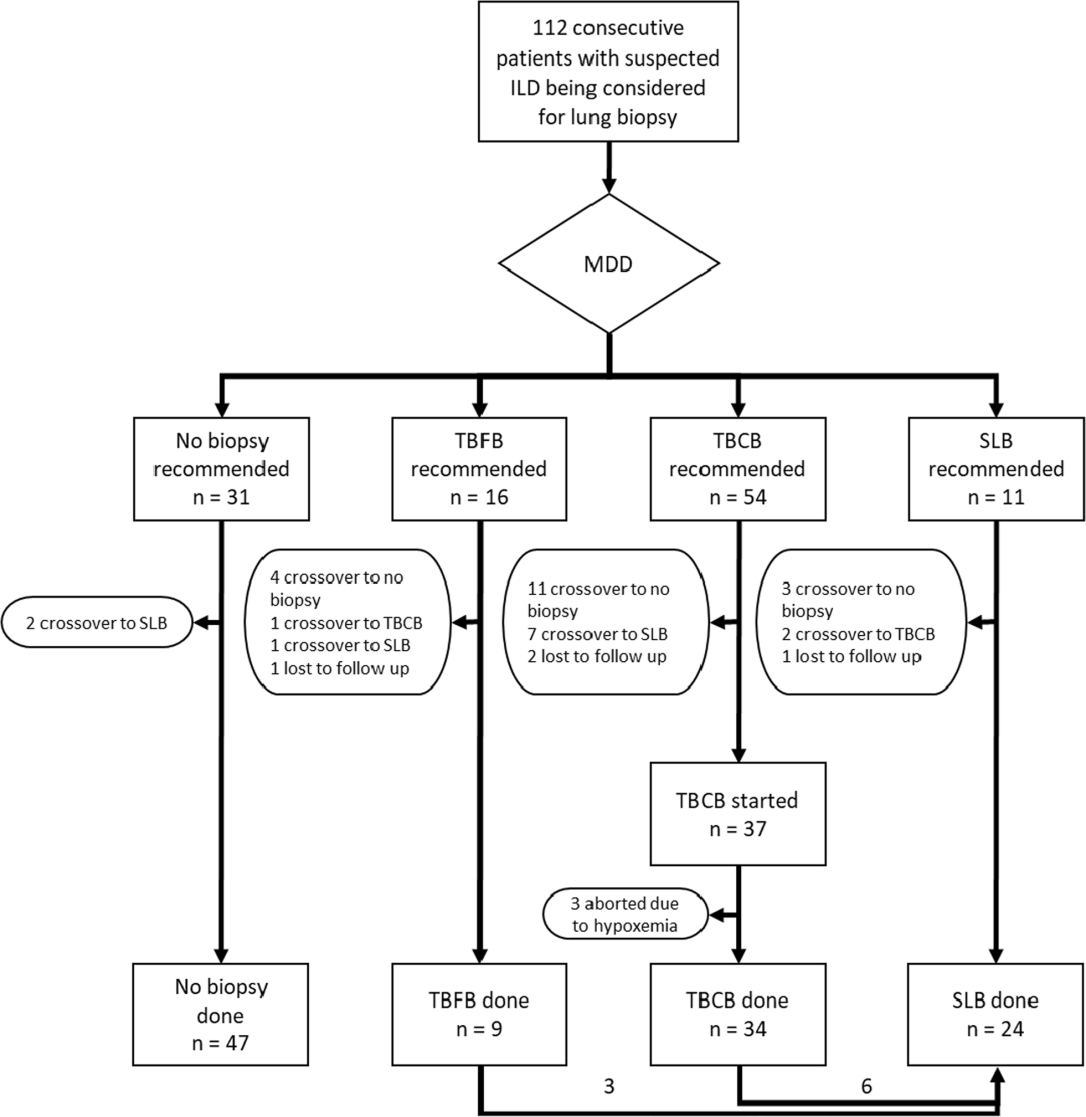
Flow diagram with study overview. ILD, interstitial lung disease; MDD, multidisciplinary discussion; TBFB, transbronchial forceps biopsy; TBCB, transbronchial cryobiopsy; SLB, surgical lung biopsy. All 4 lost to follow up were out-of-state patients who chose to follow up locally

We included 112 consecutive patients discussed in our MDD from 2017 to 2019 for a potential lung biopsy (Fig. 1).

Based on the MDD recommendation, patients were divided into 4 groups: no biopsy (n = 31), TBFB (n = 16), TBCB (n = 54) and SLB (n = 11). The overall rate of crossover between groups was 27.7% (31/112), most of them (18/32) happening from TBCB to other groups. The most common reason for crossover was patient’s choice to a different diagnostic strategy after discussion with treating physician (87%, 27/31). Other reasons were initiation of anti-platelet therapy for a coronary drug-eluting stent moving one patient from TBFB to no biopsy, acute renal failure moving one patient from TBCB to no biopsy, positive PL7 antibody moving one patient from SLB to no biopsy, and initiation of anticoagulation for pulmonary embolism moving one patient from SLB to no biopsy. Three cryobiopsy cases were aborted before the first cryobiopsy pass due to hypoxemia. In one case, hypoxemia developed after the BAL. In two other cases, hypoxemia developed during the single lung ventilation maneuver. Because TBCB was not performed, these three cases were excluded from outcome analysis. At the end of the study period, 47 patients had received no biopsy, 9 had TBFB (2 with genomic classifier), 34 had at least one pass of TBCB (5 with genomic classifier), and 24 had SLB (including 3 and 6 with non-diagnostic TBFB and TBCB, respectively).

Baseline demographics and clinical characteristics did not differ significantly between groups (Table 1).

**Table 1:** Baseline variables of 112 patients considered for lung biopsy from January of 2017 to December of 2019, stratified by MDD recommendation

Variable	Total n = 112	No biopsy n = 31	TBFB n = 16	TBCB n = 54	SLB n = 11	*p* value
Age, median (p25–p75)	67 (61–73)	66 (60–74)	70 (58–74)	67 (61–73)	64 (59–68)	0.607
Male gender, n (%)	61 (55)	19 (61)	6 (38)	32 (59)	4 (36)	0.226
Any HP exposure*, n (%)	33 (30)	10 (32)	7 (44)	13 (24)	3 (27)	0.476
Positive HP panel, n (%)	23 (21)	5 (16)	7 (44)	8 (15)	3 (27)	0.655
CTD symptoms/signs**, n (%)	13 (12)	4 (13)	2 (13)	5 (9)	2 (18)	0.736
Any CTD serologies***, n (%)	20 (18)	5 (16)	2 (13)	11 (20)	2 (18)	0.928
Pulmonary function tests						
FVC pp, median (p25–p75)	71 (57–83)	69 (57–79)	69 (54–82)	73 (57–86)	71 (58–91)	0.621
DLCO pp, median (p25–p75)	52 (42–60)	48 (35–59)	53 (42–57)	54 (45–61)	54 (40–59)	0.368
Radiology pattern, n (%)						< 0.001
Definite UIP	9 (8)	5 (16)	1 (6)	2 (4)	1 (9)	
Probable UIP	44 (39)	12 (39)	4 (25)	27 (50)	1 (9)	
Indeterminate for UIP	12 (11)	0 (0)	3 (19)	9 (17)	0 (0)	
NSIP	17 (15)	8 (26)	1 (6)	5 (9)	3 (27)	
HP	8 (7)	3 (10)	3 (19)	1 (2)	1 (9)	
OP	1 (1)	0 (0)	0 (0)	0 (0)	1 (9)	
Smoking-related ILD	7 (6)	0 (0)	2 (13)	3 (6)	2 (18)	
Sarcoidosis	1 (1)	0 (0)	0 (0)	1 (2)	0 (0)	
Unclassifiable	6 (5)	0 (0)	0 (0)	6 (11)	0 (0)	
Other	7 (6)	3 (10)	()	0 (0)	2 (18)	
Most likely diagnosis pre-biopsy, n (%)						< 0.001
IPF	22 (20)	16 (52)	1 (6)	5 (9)	0 (0)	
NSIP	7 (6)	3 (10)	0 (0)	3 (6)	1 (9)	
CTD-related ILD	2 (2)	2 (7)	0 (0)	0 (0)	0 (0)	
HP	11 (10)	4 (13)	4 (25)	3 (6)	0 (0)	
Indeterminate	64 (57)	1 (3)	11 (69)	42 (78)	10 (90)	
Other	6 (5)	5 (16)	0 (0)	1 (2)	0 (0)	

MDD, multidisciplinary discussion; TBFB, transbronchial forceps biopsy; TBCB, transbronchial cryobiopsy; SLB, surgical lung biopsy; HP, hypersensitivity pneumonitis; OP, organizing pneumonia; CTD, connective tissue disease; FVC pp, forced vital capacity percent predicted; DLCO pp, diffusing capacity for carbon monoxide percent predicted; UIP, usual interstitial pneumonia; nonspecific interstitial pneumonia; ILD, interstitial lung disease; IPF, idiopathic pulmonary fibrosis

*HP exposures: mold, birds, feathers, farm, hot tub, metalworking fluid; **CTD symptoms/signs: mechanic’s hands, digital ulcers, arthritis or morning stiffness, palmar telangiectasia, Raynaud’s phenomenon, digital edema, Gottron’s sign; ***CTD serologies: ANA, anti-centromere, rheumatoid factor, anti-CCP, anti-ds-dna, anti-SSA, anti-SSB, anti-RNP, anti-Smith, anti-Scl-70, myositis antibodies

Radiology patterns were significantly different between groups, with higher proportions of definite UIP in the “no biopsy” group and probable UIP pattern in the TBCB group. The highest proportions of nonspecific interstitial pneumonia (NSIP) were seen in the “no biopsy” and SLB groups, and most patients with a hypersensitivity pneumonitis (HP) were recommended “no biopsy” or TBFB.

### Cryobiopsy data

Most patients who had TBCB had biopsies in two segments (22/34) and in one lobe (33/34). The median freezing time was 7 s (range 5–8), and median number of passes was 4 (range 2–5). Median cryobiopsy largest diameter was 6 mm (p25–p75: 5 to 7.5 mm). Alveoli were present in 100% and pleura in 45% of the cases.

**Fig. 2:**
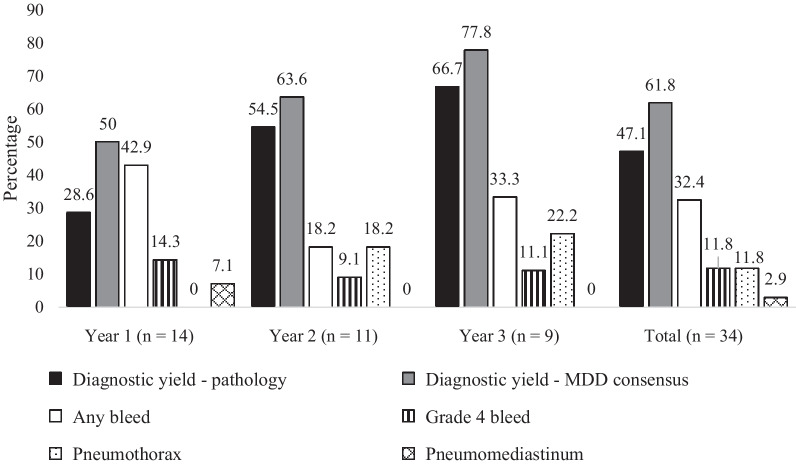
Main cryobiopsy outcomes, stratified by year of procedure. All 4 patients met criteria for grade 4 bleed because they required intubation post-bronchoscopy to control the bleeding

The number of cryobiopsy procedures decreased over time (year 1: 14, year 2: 11, year 3: 9). Diagnostic yield in both pathology and MDD consensus increased over time (Fig. 2), but the difference did not reach statistical significance (pathological yield: 4/14 in year 1, 6/11 in year 2, 6/9 in year 3, *p* = 0.161; MDD consensus yield: 7/14 in year 1, 7/11 in year 2, 7/9 in year 3, *p* = 0.412). The TBCB with non-diagnostic pathologies (n = 18) were: 3 with probable UIP (1 with patchy fibrosis, microscopic honeycombing, and lack of atypical UIP features; 2 with patchy fibrosis, fibroblast foci, and lack of atypical features), 1 indeterminate for UIP (patchy fibrosis, mild lymphoplasmacytic infiltrate, focal organizing pneumonia), 12 unclassifiable, 1 airway-centric fibrosis, and 1 normal lung.

Pneumothorax rate increased over time (year 1: 0/14, year 2: 2/11, year 3: 2/9, *p* = 0.151), while the rate of grade 4 bleed remained stable (year 1: 2/14, year 2: 1/11, year 3: 1/9, *p* = 1.000) (Fig. 2). In the 4 patients with grade 4 bleeding, values ranged from 164 to 319 k/µL for platelets, 0.7 to 1.2 mg/dL for creatinine, 13 to 34 mg/dL for blood urea nitrogen, 25.4 to 29.7 s for activated partial thromboplastin time, and 0.9 to 1 for international normalized ratio. None of the 4 patients were on antiplatelet or anticoagulant medications and 2/4 had pleura on histopathology. The first patient with grade 4 bleed developed hemoptysis in the recovery room, was reintubated, and admitted to the intensive care unit (ICU) with a bronchial blocker. He was extubated the following day. The second patient developed hemoptysis in the recovery room, was reintubated, but no active bleeding was identified. She was then extubated and discharged home. The third patient developed persistent bleeding after the fourth pass, requiring Surgicel® implantation, bronchial blocker, and admission to the ICU. He was extubated the following day. The fourth patient developed persistent bleeding after the third pass, required bronchial blocker and admission to the ICU. He was extubated the following day.

Of the 16 cryobiopsies with diagnostic pathology, most (11/16, 68.8%) had UIP, two cases (12.5%) had HP, one case had smoking-related ILD, one case had welder’s siderosis, and one case had adenocarcinoma. The prevalence of specific pathologic findings in the UIP cases were microscopic honeycombing in 80% (8/10), patchy fibrosis in 100% (10/10), fibroblast foci in 100% (10/10), and absence of atypical features of UIP in 100% (10/10). Subpleural location of the fibrosis was seen in all 5 UIP cases that contained pleura for evaluation. Ninety percent (9/10) of the UIP cases had at least 4 of the ATS 2018 guidelines’ UIP criteria. Of the 21 cryobiopsies with a consensus diagnosis in MDD, most cases (13/21, 61.9%) had IPF, three (14.3%) had HP, two (9.5%) had smoking-related ILD, one had idiopathic pneumonia with autoimmune features (IPAF), one case had welder’s siderosis, and one case had adenocarcinoma. In 6 patients with unclassifiable cryobiopsy in whom a SLB was obtained, the histopathology patterns in the SLB were definite UIP in 3, probable UIP in 1, UIP with lymphoid hyperplasia and germinal centers in 1, and smoking-related ILD in 1. In these 6 patients, the median number of passes was 4 (range 3–4), the median size was 5 mm (range 4–10 mm), alveoli were present in 6/6, but pleura was present in 0/6.

In univariate analysis, a higher number of passes was associated with a diagnostic pathology in cryobiopsies *versus* non-diagnostic cryobiopsies (median 4 [range 3–5] vs median 3.5 [range 2–4], respectively, *p* = 0.018). Other variables showed no significant association with diagnostic pathology in cryobiopsies: two segments versus one segment (diagnostic pathology in 75% vs 25% respectively, *p* = 0.297), freezing time (median 7 [range 5–8] in both diagnostic and non-diagnostic pathologies, *p* = 0.535), cryobiopsy size (median 6.5 mm in diagnostic vs 5.5 mm in non-diagnostic pathology, *p* = 0.421), presence versus absence of pleura (diagnostic pathology in 53% vs 39% respectively, *p* = 0.494). The utilization of genomic classifier was higher in TBCB patients with a consensus diagnosis versus no consensus diagnosis, but not statistically significant (23.8% vs 0% respectively, *p* = 0.132). Of note, the genomic classifier was positive for UIP in the adenocarcinoma case.

### Surgical lung biopsy data

All SLB were VATS, under general anesthesia. Of the 24 patients who had SLB, 58.3% (14/24) had diagnostic pathology (12 with UIP, one with smoking-related ILD, and one with UIP with lymphoid hyperplasia and germinal centers). Within the same group, 83.3% (20/24) had a final diagnosis (15 with IPF, three with CTD-related ILD, one with IPAF, and one with smoking-related ILD). Only 11 cases were discussed in our MDD after SLB. In the remainder 13 cases, the diagnosis was reached by the treating pulmonologist with access to the pathology results and/or individual discussion with the pathologist. This resulted in a clear pathologic classification obviating the need for MDD. The 10 non-diagnostic SLB were: 1 with chronic bronchiolitis, scattered loosely formed granulomas, and peribronchiolar metaplasia, 2 probable UIP (1 with patchy fibrosis, fibroblast foci, and minimal microscopic honeycombing; 1 with patchy fibrosis, fibroblast foci, and subpleural fibrosis), 1 indeterminate for UIP (patchy fibrosis, focal microscopic honeycombing, diffuse interstitial pneumonitis with lymphohistiocytic infiltrates, and numerous dust-laden macrophages with polarizable silica-like material), and 6 unclassifiable.

### Comparison of risks between cryobiopsy and surgical lung biopsy

TBCB patients had a longer procedure time compared to SLB, but had lower time on positive pressure ventilation, lower time on FiO_2_ of 100%, and a lower fluid balance (Table 2).

**Table 2:** Intraoperative data in cryobiopsy versus surgical lung biopsy

Variables	TBCB n = 34	SLB n = 23*	*p* value
Procedure time in mins, median (range)	50 (37–129)	44.5 (29–84)	0.015
Positive pressure time in mins, median (range)	64 (42–148)	91 (68–127)	< 0.001
Time in FiO_2_ 100% in mins, median (range)	64 (42–148)	78 (0–111)	0.042
Fluid balance in ml, median (range)	500 (0–1300)	1010 (445–2000)	< 0.001

TBCB, transbronchial cryobiopsy; SLB, surgical lung biopsy

*One patient went to have a surgical lung biopsy at another institution, so intra-operative data is missing

Table 3 shows a comparison of outcomes between TBCB and SLB. As expected, SLB patients required chest tubes routinely (100% vs 11.8% in TBCB, *p* < 0.001) and were more frequently hospitalized within 30 days compared to TBCB patients (95.7% vs 26.5%, respectively, *p* < 0.001). However, TBCB patients who were hospitalized had a longer hospital stay than SLB patients (median 2 days [range 1–4] vs median 1 [range 1–7], respectively, *p* = 0.004). The difference between mechanical ventilation requirement within 30 days was not statistically significant (8.8% in TBCB vs 0% in SLB, *p* = 0.265).

**Table 3:** Comparison of outcomes between patients who had cryobiopsies versus surgical lung biopsies

Outcomes	TBCB n = 34	SLB n = 23*	*p* value
Chest tube required, n (%)	4 (11.8)	23 (100)	< 0.001
Days with chest tube, median (range)	2 (1–2)	1 (1–7)	0.211
30-day hospitalization, n (%)	9 (26.5)	22 (95.7)	< 0.001
Length of stay in hospitalized patients, median (range)	2 (1–4)	1 (1–7)	0.004
Mechanical ventilation required, n (%)	3 (8.8)	0 (0)	0.265
Days in mechanical ventilation, median (range)	1 (1)	0 (0)	NA
30-day pneumonia, n (%)	1 (2.9)	0 (0)	1.000
30-day acute exacerbation of ILD, n (%)	0 (0)	0 (0)	NA
30-day mortality, n (%)	0 (0)	0 (0)	NA

TBCB, transbronchial cryobiopsy; SLB, surgical lung biopsy; ILD, interstitial lung disease

*One patient went to have a surgical lung biopsy at another institution, so outcome data is missing

## Discussion

In the present study, we demonstrate that the diagnostic yield of TBCB increased over time (both on pathology and MDD consensus), but the rate of grade 4 bleeding remained stable with an overall rate of 11.8%. Additionally, we show that although TBCB performed better than SLB in some variables (i.e. intra-operative duration of positive pressure ventilation and FiO_2_ of 100%, intra-operative fluid balance, and post-operative need for chest tube and hospitalization), it performed worse in others (i.e. procedure duration, length of hospital stay in hospitalized patients).

The diagnostic yield of TBCB increased over time, suggesting the existence of a learning curve. Alternatively, perhaps the MDD participants became less skeptical about the utility of TBCB overtime. A learning curve, however, has been demonstrated previously in many different bronchoscopic procedures [19–23]. Accordingly, one would expect a learning curve to also exist for TBCB. In fact, this has been previously demonstrated by Almeida and colleagues in a Portuguese cohort. In their study, the TBCB diagnostic yield improved from 74 to 90% after 50 procedures (*p* = 0.04), with a plateau being reached only after 70 procedures [24]. Their diagnostic yield was higher than ours, including in their first 25 patients (64%, compared to 47.1% in our 34 patients). Many factors could explain our lower diagnostic yield, starting from the patient population. In Almeida’s study, most patients had HP (23%) or sarcoidosis (17%), contrasting with our study where UIP was the most common radiological and pathologic finding. In addition, we reviewed every case in our MDD before the TBCB and recommended TBCB in only 48% of the cases. This most likely inserted a selection bias in our sample towards more diagnostically challenging cases. Almeida and colleagues had lower median number of passes (3 vs 4 in our cohort), lower median sample length (5.4 mm vs 6 mm in our cohort), and lower number of cases with pleura on pathology (40% vs 45% in our cohort), so those factors would not explain the difference in diagnostic yield.

Another potential explanation for our lower diagnostic yield compared to prior studies was our higher threshold to adjudicate a biopsy as “diagnostic”. The vast majority (90%) of our UIP cases on cryobiopsy had at least 4 out of the 5 ATS 2018 guidelines’ criteria for UIP. Our findings contrast with the COLDICE study, where 90.7% of the TBCB were considered diagnostic (compared to 47.1% in our study) [10]. In a secondary analysis from the COLDICE study, Cooper and colleagues showed that, in 33 patients with definite or probable UIP in the SLB, only 15.2% (5/33) had honeycombing and 9.1% (3/33) had subpleural distribution in the TBCB. Nevertheless, most of these patients (28/33) were classified as definite or probable UIP on TBCB and considered diagnostic [25, 26]. When analyzing the specific pathologic findings in all 65 patients (which included UIP and non-UIP patients), the combined presence of fibroblast foci and absence of UIP features on TBCB had sensitivity of 81.8% and specificity of 83.9% (positive and negative likelihood ratios of 5 and 0.2, respectively, with SLB as the reference standard) [25]. Although one may argue that these test characteristics are good enough to call these TBCB “diagnostic”, the ATS 2018 guidelines state that “in the absence of honeycombing, a definite diagnosis of a UIP pattern can still be made if all of the other typical features are present” [17]. This issue seems to be central to the healthy debate of how beneficial (or “diagnostic”) a cryobiopsy can be. In the largest TBCB cohort to date including 699 patients, Ravaglia and colleagues stated that “in 58% of cases the pathological diagnosis of UIP was done with high level of confidence (patchy fibrosis and fibroblastic foci with or without honey-combing and no ancillary findings against IPF)”. Moving forward, we urge for clearer pathologic definition of a “diagnostic” TBCB. Most importantly, additional prospective studies are needed demonstrating sensitivity and specificity of specific pathologic findings in TBCB compared to SLB.

One of our findings was the overall 11.8% rate (4 out of 34 TBCB patients) of grade 4 bleeding, defined as life-threatening respiratory compromise requiring intubation post-procedure. This rate remained stable throughout the three years despite an apparent improvement in diagnostic yield and presence of pleura in 45% of cases. This rate was higher than the severe bleeding rate of about 1% found in previous cohorts [11, 15]. Our higher rate was probably not explained due to a systematic difference in patient populations, since the patients with grade 4 bleeding in our study had no laboratory signs of platelet or coagulation abnormalities, and were not on antiplatelet or anticoagulant medications pre-procedure. It is unlikely, although possible, that a systematic difference in technique explained our higher bleeding rate, since we detected pleura in 45% of our samples (50% of the ones with grade 4 bleeding), higher than the 25.3% found by Ravaglia and colleagues [11]. More likely explanations for this different bleeding rate are different definitions and management strategies (which in turn are used to define severe bleed) of severe bleed in our institution compared to others. This heterogeneity in bleeding report has been demonstrated in a prior systematic review and meta-analysis [6]. Therefore, we also call for standardization of the post-bronchoscopy bleeding classification. Finally, it is likely that randomness played a role in our higher grade 4 bleeding rate, especially given our small sample size and small number of bleeding events.

Herein, we also compared TBCB with SLB outcomes. This comparison is important because those are two of the main diagnostic strategies ILD patients face in real world scenarios. The first thing to note here is that our SLB cohort performed better in some aspects compared to prior SLB cohorts. For example, we found a median length of hospital stay of 1 day (range 1–7) post SLB compared to a mean length of 6.1 days (range 3–48) in a Italian retrospective cohort study [3]. In that study, which included 150 SLB cases, the rate of acute exacerbation of IPF was 3.3%, pneumonia was 2%, and 60-day mortality was 2.7% after SLB [3]. The 30-day incidence of those adverse events in our SLB cohort was zero. In a meta-analysis including 2665 SLB patients, surgical mortality was 2.3% (1.3–3.6%) and mean length of hospital stay was 3.8 days (range 2.8 to 5.5 days) [4]. The second important thing to note is that, in our cohort study, the SLB group had a shorter procedure time compared to the TBCB group (median 44.5 min vs 50 min, respectively). However, patients in the TBCB experienced less time in positive pressure, less time on 100% FiO_2_, and a lower fluid balance. This calls for an RCT comparing TBCB versus SLB as two separate diagnostic strategies in ILD.

Our study has several limitations. First, since this was not an RCT, allocation to the different diagnostic strategies was not random. We allocated patients to each group prospectively during our MDD, which certainly increases the risk of bias and decreases the internal validity of our comparison. In addition, the possibility of a learning curve in our cryobiopsy arm jeopardizes even further our ability to compare those two groups. Our results comparing TBCB and SLB can only be seen as hypothesis-generating and require validation in a future RCT. Second, our small sample size increases the risk of random error. This is certainly a limitation especially given the larger cohort studies available in the literature, and it limits the conclusions we can draw from our data. Third, our single center study limits its external validity. Characteristics of our institution and practice (e.g. quaternary academic medical center, highly specialized physicians, definition and management of severe endobronchial bleed) may not be applicable to other places. Finally, there was a high rate of crossover between groups, mainly from TBCB to other groups. This was largely due to patient-centered discussions regarding the risks, benefits, and alternatives to the MDD recommendations. Nonetheless, it is certainly a limitation that threatens the internal validity of our findings.

In conclusion, our prospective cohort study adds another word of caution to the healthy debate around cryobiopsy in ILD. Despite showing a possible learning curve in both pathologic and MDD diagnostic yields, the rate of grade 4 bleed (11.8%) remained unacceptably high to our group. In highly specialized centers like ours, other diagnostic strategies may be better for patients than TBCB. Our conclusions are in concordance with the ATS 2018 guidelines that recommend that experienced centers in TBCB continue to work towards an optimal balance between risks and benefits [17]. Once or if this balance is achieved, educational interventions will be necessary to study how to best overcome the learning curve in non-experienced centers. And finally, TBCB will need to be tested in a randomized fashion against SLB comparing patient-important outcomes, which will give ILD physicians valuable data to share with patients.

## Supplementary Information

**Additional file 1.** Deidentified RedCap data.

**Additional file 2.** RedCap data dictionary.

## Data Availability

All data generated or analyzed during this study are included in this published article as a Additional files 1 and 2 exported from RedCap®.
